# A Customized Novel Blocking ELISA for Detection of Bat-Origin Swine Acute Diarrhea Syndrome Coronavirus Infection

**DOI:** 10.1128/spectrum.03930-22

**Published:** 2023-06-05

**Authors:** Liyan Cao, Xiangyu Kong, Xiangtong Li, Xuepeng Suo, Yueyue Duan, Cong Yuan, Yu Zhang, Haixue Zheng, Qi Wang

**Affiliations:** a Institute of Urban Agriculture, Chinese Academy of Agricultural Sciences, Chengdu, China; b State Key Laboratory of Veterinary Etiological Biology, Lanzhou Veterinary Research Institute, National Foot and Mouth Diseases Reference Laboratory, Chinese Academy of Agricultural Sciences, Lanzhou, China; c Chengdu National Agricultural Science and Technology Center, Chengdu, China; University of California, Davis

**Keywords:** SADS-CoV, blocking ELISA, antibody detection, bat-origin swine coronavirus, customized a novel blocking ELISA, SADS-CoV infection

## Abstract

Swine acute diarrhea syndrome coronavirus (SADS-CoV) is a newly discovered emerging alphacoronavirus. SADS-CoV shares over 90% genome sequence identity with bat alphacoronavirus HKU2. SADS-CoV was associated with severe diarrhea and high mortality rates in piglets. Accurate serological diagnosis of SADS-CoV infection is key in managing the emerging SADS-CoV. However, thus far there have been no effective antibody-based diagnostic tests for diagnose of SADS-CoV exposure. Here, monoclonal antibody (MAb) 6E8 against SADS-CoV N protein accurately recognized SADS-CoV infection. Then, MAb 6E8 was utilized as a blocking antibody to develop blocking ELISA (bELISA). We customized the rN coating antigen with concentration 0.25 μg/mL. According to receiver operator characteristic curve analysis, the cutoff value of the bELISA was determined as 38.19% when the max Youden index was 0.955, and specificity was 100%, and sensitivity was 95.5%. Specificity testing showed that there was no cross-reactivity with other serum positive swine enteric coronaviruses, such as porcine epidemic diarrhea virus (PEDV), transmissible gastroenteritis virus (TGEV), porcine deltacoronavirus (PDCoV), porcine rotavirus (PoRV), and porcine sapelovirus (PSV). In conclusion, we customized a novel and high-quality blocking ELISA for detection of SADS-CoV infection, and the current bELISA will be linked to a clinical and epidemiological assessment of SADS-CoV infection.

**IMPORTANCE** SADS-CoV was reported to be of high potential for dissemination among various of host species. Accurate serological diagnosis of SADS-CoV infection is key in managing the emerging SADS-CoV. However, thus far there have been no effective antibody-based diagnostic tests for diagnose of SADS-CoV exposure. We customed a novel and high-quality bELISA assay for detection of SADS-CoV N protein antibodies, and the current bELISA will be linked to a clinical and epidemiological assessment of SADS-CoV infection.

## INTRODUCTION

Swine acute diarrhea syndrome coronavirus (SADS-CoV) was first isolated from suckling piglets with diarrhea in China in 2017 (1). SADS-CoV, also named swine enteric alphacoronavirus (SeACoV) (2) or porcine enteric alphacoronavirus (PEAV) (3), causes acute diarrhea, vomiting, dehydration, and eventually death due to the rapid weight loss of newborn piglets. SADS-CoV is a member of the genus *Aiphaconavirus* in the subfamily *Orthocoronavirinae* of the family *Coronaviridae* with a genome of appropriately 27 kb in length. SADS-CoV is closely related with bat coronavirus HKU2, the nucleotide identities were more than 90% and contained 5′ untranslated region (UTR), open reading frame 1a/1b (ORF1a/1b), spike (S), nonstructural protein 3 (NS3), envelope (E), membrane (M), nucleocapsid (N), nonstructural protein 7a (NS7a), and 3′ UTR (4).

The clinical and pathological symptoms of SADS-CoV are similar to other porcine enteric coronaviruses, such as porcine epidemic diarrhea virus (PEDV) (5), transmissible gastroenteritis virus (TGEV) (6), and porcine deltacoronavirus (PDCoV) (7), making it difficult to differentiate among them. Pigs were commonly coinfected with more than one enteric pathogen, and the clinical outcome was often more severe (8, 9). There is no vaccine or clinical approved drug to prevent and cure SADS-CoV. Therefore, to prevent SADS-CoV infection, it is very important to develop a rapid and reliable detection for SADS-CoV. So far, the diagnosis of SADS-CoV infection mainly relies on viral nucleic acid detection by using the methods of TaqMan-based real-time RT-PCR assay (10), real-time reverse transcription loop-mediated isothermal amplification method (RT-LAMP) (11), SYBR green-based real-time RT-PCR assay (12), TaqMan-probe-based multiplex real-time PCR (13, 14), microfluidic-RT-LAMP chip (15), a novel reverse transcription droplet digital PCR assay (16), and CRISPR-Cas12a combined with multiplex RT-LAMP (17). Serological diagnosis of SADS-CoV was reported rarely. The enzyme-linked immunosorbent assay (ELISA) is the most common method of serological assay. The ELISA uses a luciferase immunoprecipitation system based on the S1 protein (4), and indirect ELISA (iELISA) based on the S protein (18) and virion (19) were developed and have been used to detect the changes of antibodies levels of SADS-CoV.

Based on the characteristics of high immunogenicity and conservation, N protein is an ideal antigen for development of serological methods. In this study, for the first time, a blocking ELISA (bELISA) for detecting of pig anti-SADS-CoV serum antibodies was developed using the purified N protein as coating antigen and anti-N MAb as the blocking antibody. The N protein was fused with His tag and purified by Ni-chelating affinity chromatography, then immunized mouse to produce monoclonal antibody (MAb). Five MAbs against the SADS-CoV N protein were obtained, and one MAb (entitled 6E8) had a good reactivity with SADS-CoV and was used as blocking antibody to develop the bELISA. The established bELISA in our study has a favorable application for detection of anti-SADS-CoV antibodies in clinical samples.

## RESULTS

### Expression and purification of rN protein.

**FIG 1:**
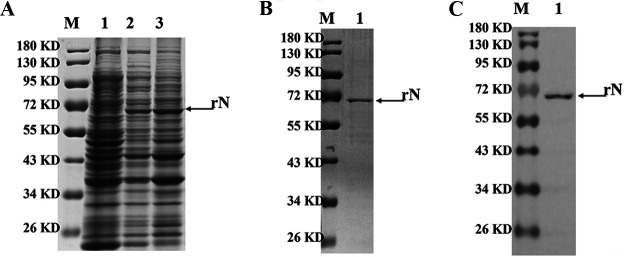
Purification and identification of rN protein. (A) The solubility of rN protein analyzed by SDS-PAGE. Lane 1, noninduced rN protein; lane 2, suspension of the bacterial lysate; lane 3, precipitate of the bacterial lysate. (B) Purification of rN protein analyzed by SDS-PAGE. Lane 1, purified rN protein. (C) Western blot analysis. The purified rN protein could react with the positive pig serum of SADS-CoV. Lane M, protein molecular weight marker.

The recombinant plasmid pET32-N with a His tag was transformed into E. coli host cells. SDS-PAGE analysis showed that part of rN protein (approximately 67 kDa) was expressed in soluble form (Fig. 1A). After being purified using Ni-NTA, the rN protein was identified by SDS-PAGE, and the immunoreactivity of the rN protein was confirmed by Western blotting. Results showed that the rN protein had a high purity (Fig. 1B), which could produce a strong reaction with the SADS-CoV positive serum (Fig. 1C).

### Production MAb against SADS-CoV N protein.

**FIG 2:**
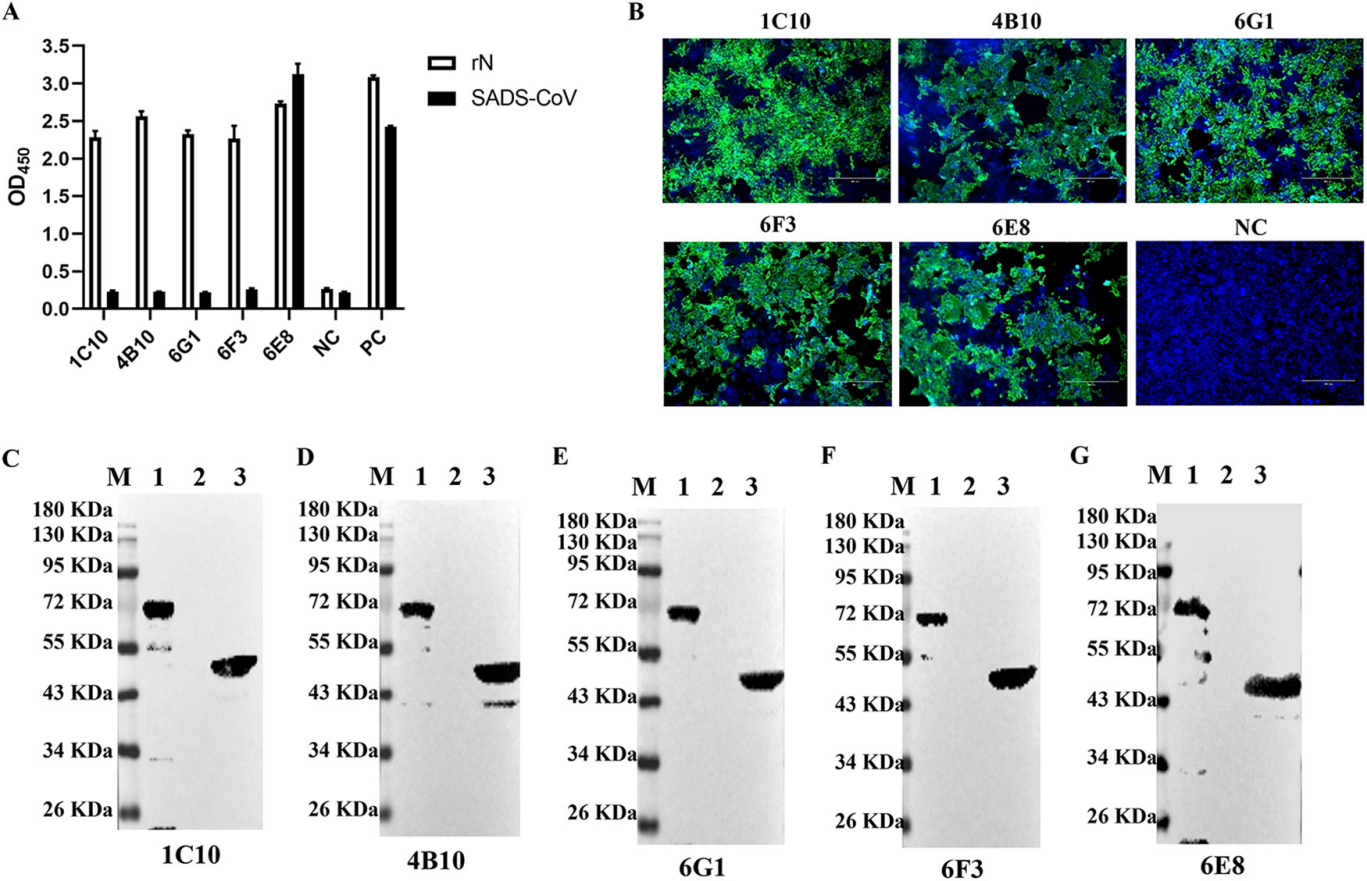
Reactivity of anti-N MAb. (A) iELISA. The supernatants of hybridoma were added into coating the rN protein or SADS-CoV ELISA plates. The supernatant of SP2/0 cells were (as negative control, NC) and mouse polyclonal antibody to SADS-CoV N protein (as positive control, PC) were used as control. (B) IFA assays. The MAb 1C10, 4B10, 6G1, 6F3 and 6E8, and SP/20 cell supernatant as primary antibody were stained SADS-CoV infected cells. The supernatant of SP2/0 cells were used as negative control (NC). The nuclei were stained with DAPI. (C-G) Western blot. The purified rN and the lysates of SADS-CoV-infected or mock-infected cells were subjected to Western blot analysis with the MAb 1C10 (C), 4B10 (D), 6G1 (E), 6F3 (F) and 6E8 (G). Lane M, protein molecular weight marker; lane 1, the purified rN protein; lane 2, SADS-CoV mock-infected cells; lane 3, SADS-CoV-infected cells.

The purified rN was used as an immunogen to immunize mouse and five specific MAbs against SADS-CoV N protein (designated 1C10, 4B10, 6G1, 6F3, and 6E8) was obtained by hybridoma cell fusion technique. The reactivity and specificity of MAbs were identified by IFA, iELISA, and Western blot. By iELISA, the five MAbs could react with the rN protein, while only the MAb 6E8 could react with SADS-CoV (Fig. 2A). As shown in Fig. 2B, all MAbs recognized the native N protein in cells infected with SADS-CoV, and the supernatant of SP2/0 cells (NC group) had no fluorescent. Furthermore, the results of Western blot showed the five MAbs specifically recognized with the purified rN protein, as well as the native N protein in cells infected with SADS-CoV (Fig. 2C to G). Combined with the results of IFA, iELISA, and Western blot, we selected the MAb 6E8 to develop the bELISA.

### Establishment of the bELISA.

**FIG 3:**
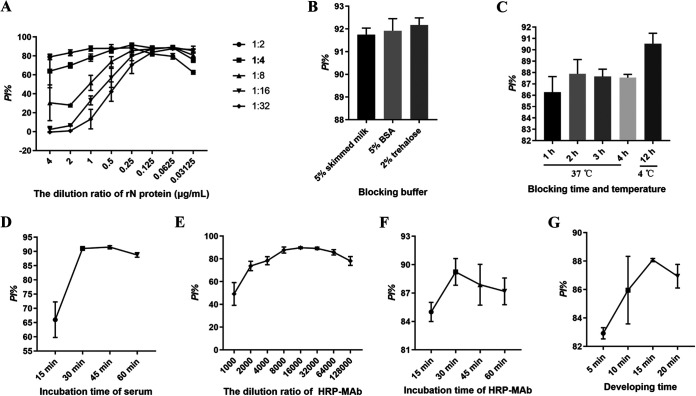
Optimal conditions for the bELISA. (A) Different concentrations of coating antigen rN (4, 2, 1, 0.5, 0.25, 0.125, 0.0625 and 0.03125 μg/mL) and dilutions of serum samples (1:2, 1:4, 1:8, 1:16, and 1:32) were tested by checkerboard. The blocking buffer (B) and the optimal blocking time (C) were evaluated. 5% skimmed milk, 5% BSA and 2% trehalose were used as the blocking buffer, and the blocking conditions were under 37°C for 1–4 h, or 4°C for 12 h. (D) Influence of detection serum reaction time. Serum samples was diluted 1:4 in PBST, and incubated 37°C for 15, 30, 45, and 60 min, respectively. Determination of the optimal dilution ratio (E) and reaction time (F) of HRP-MAb. The HRP-MAb was serial diluted (1:1000–1:128000), and incubation time was 37°C for 15, 30, 45, and 60 min, respectively. (G) Optimum the developing time. TMB was incubated with 37°C for 5, 10, 15, and 20 min, respectively, and then stopped in 2 M H_2_SO_4_. The PI value was calculated, and the highest PI value was the optimal work conditions of the bELISA.

The purified rN as an antigen was coated ELISA, and the purified MAb 6E8 as a blocked antibody was labeled with HRP. Using a checkerboard titration test, the concentration of the rN antigen 0.25 μg/mL and serum sample diluted in 1:4 were the best pair that produced the highest PI value (Fig. 3A). Furthermore, it was found that 2% threhalose was the best blocking solution (Fig. 3B), and 4°C for 12 h was the optimal blocking time (Fig. 3C). As shown in Fig. 3D, the optimal reaction time of serum samples was 45 min. Next, the optimal dilution ratio of HRP-MAb and the effect of incubation time at 37°C was assessed. As shown in Fig. 3E and F, the optimal dilution ratio and reaction time of HRP-MAb were 1:16000 and 30 min, respectively. Finally, effects of the developing time were observed, and the experimental results suggested that the PI value was highest at 37°C for 15 min in darkness (Fig. 3G).

### Determination of the cutoff value of the bELISA.

**FIG 4:**
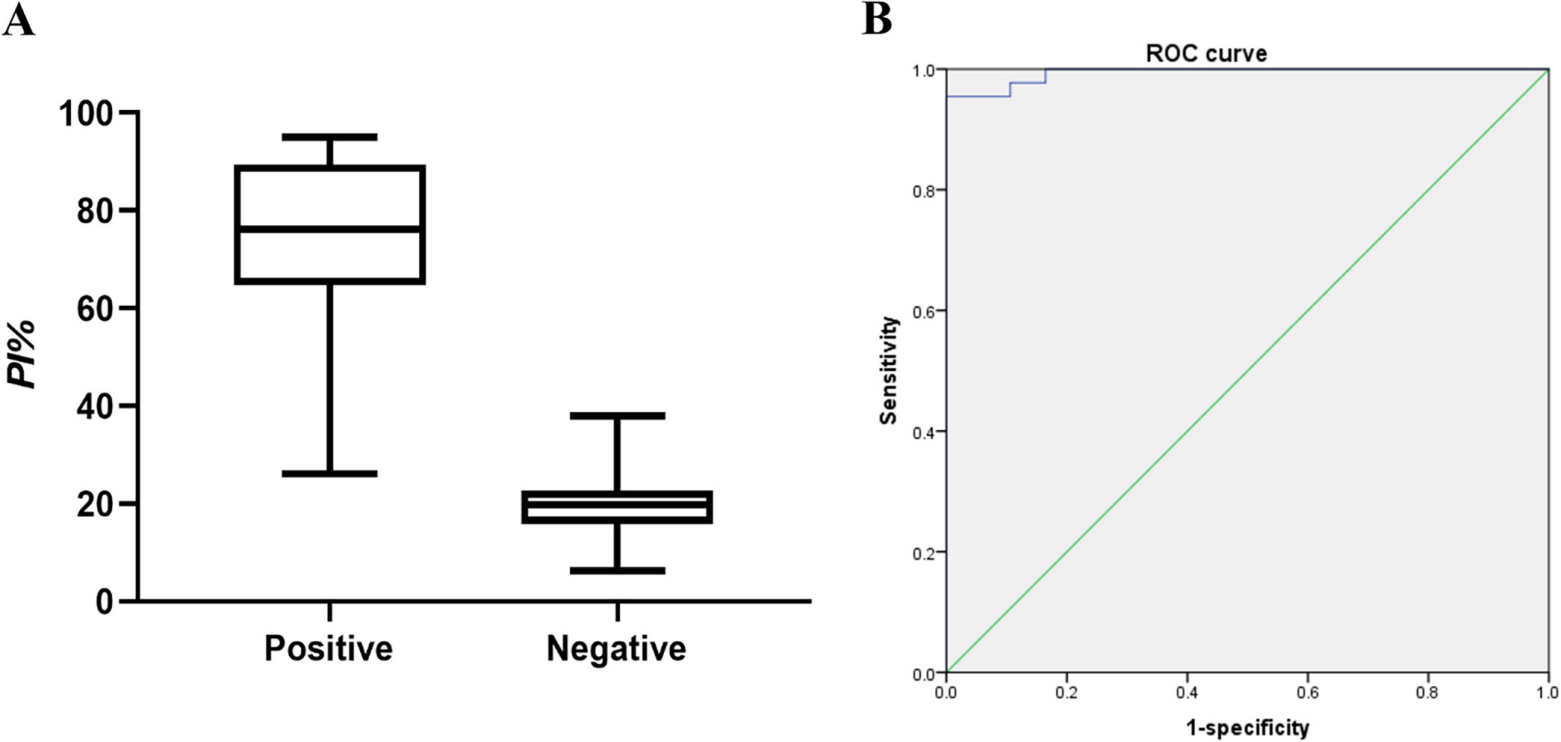
Determine the cutoff value. (A) 129 serum samples (44 positive and 85 negative) were subjected the optimal the bELISA. PI values were calculated. (B) ROC curve was drawn by SPSS 23. According to the ROC curve, the cutoff value was 38.19%.

To determine cutoff value, 129 serum samples (44 positive and 85 negative) were evaluated under the above optimal conditions (Fig. 4A). The PI values of 129 serum samples were used to estimate the sensitivity and specificity by drawing the ROC. According to ROC analysis, when the cutoff value was 38.19%, the Youden index reached maximum (0.955), and the sensitivity was 95.5%, and the specificity was 100% (Fig. 4B).

### Specificity, sensitivity, reproducibility of the bELISA.

**FIG 5:**
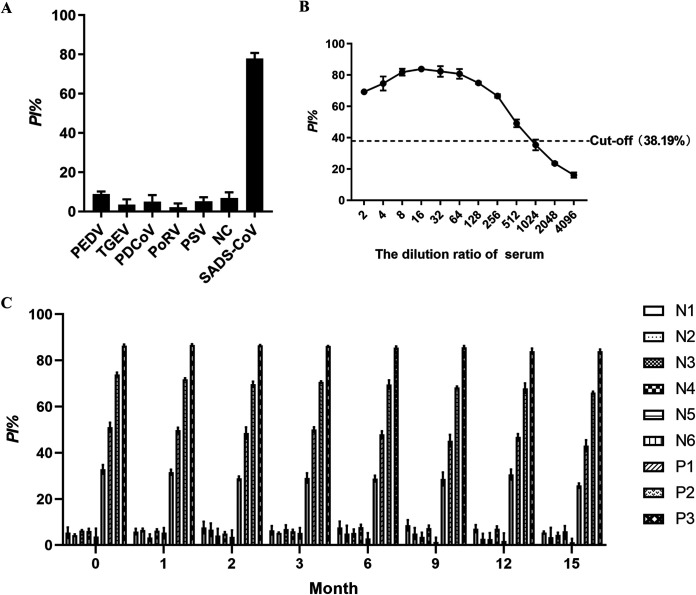
Validation of the bELISA. (A) Specificity. The sera against SADS-CoV, PEDV, TGEV, PDCoV, PoRV and PSV were used to evaluate specificity of the bELISA. (B) Sensitivity. Serum samples were diluted 1:2 to 1:4096 and used the bELISA to evaluate the sensitivity. (C) Stability. Three batches of the bELISA plates were stored at 4°C. N1-6 and P1-3 were detected using bELISA plates at 0, 1, 2, 3, 6, 9, 12 and 15 months, respectively. P1-3 were positive serum samples for SADS-CoV, and N1-6 were negative controls.

To determine the specificity of the bELISA, SADS-CoV positive, and negative serum samples, as well as samples positive for other swine enteric coronaviruses (i.e., PEDV, TGEV, and PDCoV), PoRV and PSV were tested. The test results showed that the PI value was 77.93% for SADS-CoV-positive sera and SADS-CoV-negative and PEDV, TGEV, PDCoV, PoRV, and PSV-positive sera were less than cutoff value, suggesting that the bELISA for the detection of SADS-CoV antibody had good specificity and no cross-reactivity with other swine enteric coronaviruses and diarrhea related pathogens PoRV and PSV (Fig. 5A).

To determine the sensitivity of the bELISA, positive and negative standard pig serum sample for SADS-CoV were double diluted to 1:2, 1:4, 1:8, 1:16, 1:32, 1:64, 1:128, 1:256, 1:512, 1:1024, 1:2048, and 1:4096, respectively. As shown in Fig. 5B, the PI value was >38.19%, when the samples diluted 512. The data suggested that the bELISA is a sensitive method.

The intra-assay reproducibility was calculated by analyzing six positive (P1-6) and three negative (N1-3) serum samples for SADS-CoV for a total of 5 times in a single batch. Moreover, three batches of the bELISA were analyzed to confirm inter assay reproducibility. As shown in Table 1, the intra assay CV and the inter assay CV were <10%, suggesting good reproducibility of the bELISA.

**TABLE 1:** Inter and intra reproducibility of the bELISA^*a*^

Repeatability assay	Serum	Times	Average	SD	CV%
Inter-assay	P1	5	84.51	0.23	0.27
	P2	5	79.28	0.75	0.95
	P3	5	71.94	1.07	1.49
	P4	5	60.83	0.61	1.00
	P5	5	48.63	0.80	1.65
	P6	5	53.54	0.75	1.40
	N1	5	13.44	1.29	9.57
	N2	5	9.23	0.51	5.48
	N3	5	30.61	1.15	3.75
Intra-assay	P1	5	84.59	1.46	1.72
	P2	5	78.35	1.12	1.42
	P3	5	72.18	2.52	3.49
	P4	5	61.57	2.15	3.49
	P5	5	49.08	1.03	2.10
	P6	5	54.11	1.91	3.52
	N1	5	9.49	0.65	6.86
	N2	5	13.76	1.02	7.40
	N3	5	30.98	2.44	7.88

a SD, standard deviation; CV, coefficient of variation.

To determine the stability of the bELISA, the ELISA plates were stored at 4°C for 0 to 15 months. The serum samples of N1-6, and P1-3 were detected at 0, 1, 2, 3, 6, 9, 12, and 15 months, respectively. As shown in Fig. 5C, the PI values of N1-5 were below 10%, and N5 was 32.80%, and P1-3 were 51.03%, 73.96%, and 86.41% at initial time. After 15 months, PI value has a slightly decreasing, but it can still distinguish between positive and negative, suggesting that the bELISA for SADS-CoV antibody detection had high specificity that kept for 15 months. Combined with the specificity and sensitivity assay, the data showed that the bELISA for SADS-CoV antibody detection had good stability.

### Comparison of the bELISA and IFA.

**FIG 6:**
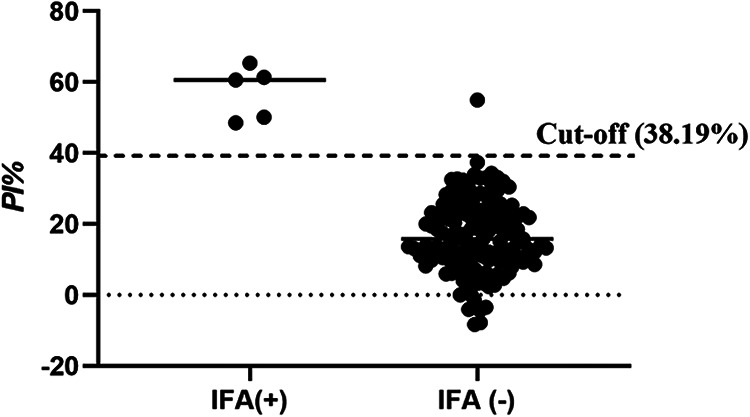
Efficiency of bELISA was evaluated by clinical serum samples. 150 of clinical pig serum samples were collected and subjected to bELISA and IFA assays.

A total of 150 pig serum samples were collected from a pig farm in Gansu Province and tested by the bELISA (Table S1 in the supplemental material) and IFA (Fig. S1 in the supplemental material). As shown in Fig. 6, 6 of the 150 serum samples were determined to be positive by using bELISA, whereas 5 samples were positive by IFA. Of the total 150 serum samples, 5 were positive and 144 were negative, as determined using these two methods. The accuracy of these two detection methods was 99.33% (Table 2). In addition, the kappa value was 0.90, suggesting a high level of agreement between the bELISA and IFA methods.

**TABLE 2:** Consistency between the bELISA and IFA^*a*^

Blocking ELISA	IFA		Total	Coincidence rate
	Positive	Negative		
Positive	5 (a)	1 (b)	6 (a + b)	99.33%
Negative	0 (c)	144 (d)	144 (c + d)	
Total	0 (a + c)	145 (b + d)	150 (n)	
Kappa	0.90			

a The coincidence rate was calculated as ([a + d]/n) × 100. The K value was calculated as follows: P_A_ = (a + d)/n; P_e_ = [(a + b) (a + c) + (c + d) (b + d)]/n^2^; Κ = (P_A_-P_e_)/(1-P_e_). Κ ≥ 0.75 means good consistency; 0.75 > Κ ≥ 0.4 means moderate consistency; Κ < 0.4 means poor consistency.

## DISCUSSION

In recent years, the emergence of new or reemerging coronavirus has posed a great threat to human and animal health, which has caused high attention to coronavirus prevention and control. For example, severe acute respiratory syndrome coronavirus 2 (SARS-CoV-2) is a zoonotic infection, newly discovered in Wuhan, Hubei province of China in 2019, because of its prevalence and mortality causing a global pandemic (20, 21). SADS-CoV as an experiment object in this study, which is also a newly discovered coronavirus in Guangdong province of China in 2017 that could infect all age pigs, with a mortality rate up to 90% in piglets 5 days or younger (1, 2). After May 2017, the epidemic of SADS was controlled. While on February 2019, there was a large-scale SADS pandemic in southern China (22). In 2020, from 1 July to 30 September, it was reported that a total 300 serum samples were obtained from commercial growing pigs on different farms with reported diarrhea outbreaks from 11 provinces (Shanxi, Yunnan, Guangdong, Jiangxi, Henan, Hubei, Hebei, Hunan, Qinghai, Anhui, and Shanxi) in China. Among these 300 serum samples, 81.7% (245/300) of serum samples were positive for SADS-CoV (18). These results showed that these places had existing SADS-CoV infection. In this study, we detected 5 of the 150 serum samples were SADS-CoV positive in pig farms from Gansu province. However, there are no commercially available vaccines and diagnostic kits for SADS-CoV at present.

SADS-CoV is a coronavirus, and coronavirus has four main structural proteins: nucleocapsid (N), spike (S), membrane (M) and envelope (E). The S protein is highly immunogenic since it is located on the viral surface (23). The N protein plays an important role in the transcription and replication of viral RNA, packaging the encapsidated genome into virions (24). In addition, the N protein is abundantly expressed during infections and also has high immunogenic activity (25). Therefore, both N and S protein could be potential targets for the antibody-based detection of SADS-CoV. Recent studies showed that N protein reacted with most of patient sera with coronavirus infection and serum samples from acute phase of patients (5 to 10 days after coronavirus infection). In contrast, the serum samples from acute phase of patients with coronavirus infection did not respond to S protein, suggesting that antibodies to N protein developed earlier than S protein-specific antibodies (25). Recent studies showed that N protein reacted with most patient sera with coronavirus infection and serum samples from acute phase of infection (5 to 10 days after coronavirus infection). In contrast, the serum samples from acute phase of coronavirus infection did not respond to S protein, suggesting that antibodies to N protein developed earlier than S protein-specific antibodies (25). Therefore, we developed and evaluated a bELISA for detection of antibodies against SADS-CoV N protein. SADS-CoV N protein was highly expressed using the prokaryotic expression system and purified by Ni-chelating affinity chromatography (Fig. 1). The bELISA used the purity rN protein as a coating antigen, and the optimum coating concentration was 0.25 μg/mL (Fig. 2A). The blocking MAb against SADS-CoV N protein was obtained by the hybridoma cell fusion technique. IFA and Western blot analysis showed that the MAbs (1C10, 4B10, 6G1, 6E8, and 6F3) could recognize the N protein in cells infected with SADS-CoV (Fig. 2B to G). Among these five MAbs, 6E8 showed much higher binding capability to SADS-CoV by iELISA (Fig. 2A), suggesting that the MAbs have a different affinity and epitope specificity. Thus, the MAb 6E8 was selected as detection antibody and labeled with HRP. The optimal dilution of serum samples and HRP-6E8 were 1:4 and 1:16000, and the optimal immunoreaction times were 45 min and 30 min at 37°C, respectively (Fig. 3).

Currently, there are six CoVs that can infect pigs, including TGEV, porcine respiratory coronavirus (PRCV) (26), PEDV, PDCoV, SADS-CoV, and porcine hemagglutinating encephalomyelitis virus (PHEV) (27). TGEV, PEDV, PDCoV, and SADS-CoV are the pathogens of porcine enteric coronavirus disease with similar clinical signs and pathogenesis (28–30). PoRV and PSV are also the important diarrhea related pathogens in pigs. The bELISA for the detection of SADS-CoV antibody in this study had high specificity and no cross-reactivity with TGEV, PEDV, PDCoV, PoRV, and PSV (Fig. 5A).

Indirect ELISA (iELISA) is a technique that uses a two-step process for detection, whereby a primary antibody specific for the antigen binds to the target, and a labeled secondary antibody against the host species of the primary antibody binds to the primary antibody for detection specific antibodies of viral infection. Blocking ELISA (bELISA) in our study is developed based on specific monoclonal antibodies. bELISA efficiently decreases nonspecific reactions and improves the specificity of detection specific antibodies of SADS-CoV infection. Generally, the analytical characterization of the development method needs to be compared with other commercial kits; however, there is no commercially available test kit for SADS-CoV at present. Thus, the serum samples were determined by IFA assay as standards for antibody status. The total coincidence rate and kappa value for the agreement between the bELISA and IFA were 99.33% and 0.90, by detection 150 clinical serum samples, suggesting that bELISA is a reliable method for detection of antibodies against SADS-CoV.

To the best of our knowledge, this is the first report of a bELISA for detection of SADS-CoV N protein antibodies. We reported that the method of iELISA was used to detect SADS-CoV antibodies. As is well known, the purity of coating antigen of iELISA is required to be relatively high to avoid the nonspecific binding artifacts. The bELISA is developed basing on the MAb, which could decrease nonspecific reactions and improve the specificity of detection of antibodies in serum samples. Preliminary evaluation of our bELISA demonstrated a sensitivity of 95.5%, specificity of 100%; the intraassay and interassay variability are <10% and stability over a 15 month period. The bELISA has a favorable application prospect for the development of a diagnostic test for the sero-epidemiology and outbreak investigation of SADS-CoV.

## MATERIALS AND METHODS

### Cells, viruses, and serum samples.

Huh7 cells were cultured in Dulbecco minimum essential medium (DMEM) supplemented with 10% fetal bovine serum (FBS) and incubated under an atmosphere of 5% CO2 at 37°C. SADS-CoV strain GDS04 (GenBank Accession No. MF167434.1) was isolated and kindly provided by the professor of Sun Yat-Sen University of Yongchang Cao (1). Huh 7 cells were used to propagate SADS-CoV as described previously (19). Positive serum samples for SADS-CoV, PEDV, TGEV, PDCoV, porcine rotavirus (PoRV), and porcine sapelovirus (PSV) and healthy pig serum were kept in our laboratory.

### Expression and purification of the recombinant N (rN).

The complete N (1128 bp) gene was amplified from SADS-CoV strain GDS04 using the specific primers (F: 5′-CGCGGATCCATGGCCACTGTTAATTGG-3′, and R: 5′-CCGCTCGAGCTAATTAATAATCTCATC-3′), and the PCR production was inserted into the pET32a vector at restriction enzyme sites for BamHI and XhoI. The recombinant plasmid pET32a-N transformed into *Escherichiacoli* (E. coli) BL21(DE3) and induced with 1 mM isopropyl-d-thiogalactopyranoside (IPTG) at 16°C for 16 h in Luria-bertni medium (LB). The E. colicells were harvested by centrifugation at 10,000 r/min for 10 min, and resuspended in phosphate-buffered saline (PBS). After ultrasonication on ice for 10 min, the bacterial lysate was centrifuged at 4°C, 12,000 r/min for 10 min, and collected the supernatant. The supernatant was purified through Ni-chelating affinity chromatography, and the purity was identified by SDS-PAGE, and the immunoreactivity was detected by Western blotting.

### Production and purification of N MAb.

The purified rN protein immunized the 6- to 6-week-old female BALB/c mice (Lanzhou, China). The purified rN protein (30 μg/mouse) was mixed with the equal volume of Freund’s complete adjuvant (Sigma-Aldrich, St. Louis, MO, USA) and injected subcutaneously in multiple spots on the belly. At 2-week intervals, booster immunization was given by using the purified rN protein (30 μg/mouse) mixed with equal volume of Freund’s incomplete adjuvant (Sigma-Aldrich, St. Louis, MO, USA) as following immunization antigen. After 4 immunizations, spleen cells were isolated and fused with mouse myeloma SP2/0 cells as previously described (31).The antibodies secreted by hybridomas were detected by indirect ELISA (iELISA) and Immunofluorescence assay (IFA). Positive clones were subcloned 3 times by limiting dilution and injected into Freund’s incomplete adjuvant-treated BALB/c mice to obtain ascetic fluid. Immunoglobulin G (IgG) from MAb were purified using protein Gsepharose (GE Healthcare, Chicago, IL, USA).

### iELISA.

The purified rN protein (100 ng/well) or the culture supernatants of SADS-CoV-infected Huh7 cells (1:1) were diluted in carbonated coating buffer (pH 9.6), and 50 μL was used to coat ELISA plates at 4°C overnight. The plates were washed 4 times with PBST (phosphate-buffered saline [PBS] containing 0.05% Tween 20). The plates were then blocked with 2% trehalose in PBST at 4°C overnight, and then washed with PBST 4 times. The rN immunized mouse serum (diluted 1:100 in PBST), the supernatants of hybridoma or SP2/0 cells were added into ELISA plates. The plates were incubated for 30 min at 37°C, then washed 4 times, and incubated with HRP-conjugated goat anti-mouse IgG (Abcam, Cambridge, MA, USA) diluted at 1:20000 in PBST at 37°C for 30 min. The plates were washed again, and developed with TMB, stopped by using 2M H_2_SO_4_. The absorbance was measured at 450 nm. The rN immunized mouse serum was used as positive control (PC), and the supernatant of SP2/0 cells as negative control (NC).

### IFA.

To detect the reactivity of MAb or pig serums with SADS-CoV, IFA was used. Huh7 cells were seeded on a 96-well plate at a density of 1 × 10^4^ cells/well, grown in monolayers, and then infected with SADS-CoV at a multiplicity of infection (MOI) of 0.01 for 36 h. The cells were washed three times with PBS, fixed with 4% paraformaldehyde for 30 min, and permeabilized with 0.1% Triton X-100 for 10 min. After washing 3 times with PBS, cells were incubated with the supernatants of hybridoma or SP2/0 cells, or pig serums (dilution, 1:500) at 37°C for 1 h. Cells were washed with PBS, and then incubated with a FITC-conjugated anti-mouse or pig IgG secondary antibody (Sigma-Aldrich, St. Louis, MO, USA, dilution, 1:100) at 37°C for 30 min. Cells were washed with PBS again, and the nuclei were stained with 4′,6-diamidino-2-phenylindole (DAPI). Cells were analyzed with the fluorescence microscope (AMG EVOS F1; Advanced Microscopy Group, Mill Creek, WA, USA).

### Western blot.

Huh7 cells were seeded into 6-well plate at a density of 1 × 10^6^ cells/well for 24 h, and infected or mock-infected with SADS-CoV at an MOI of 0.01 for 36 h. Cells were harvested and lysed with RIPA lysis buffer containing 1 mM PMSF. To identify the reactivity of MAbs, the purified rN protein and the native N protein of SADS-CoV infected or mock-infected cells were separated by SDS-PAGE and transferred to nitrocellulose (NC) membranes. The membranes were blocked with 5% (wt/vol) skim milk powder in PBST at room temperature for 1 h. Subsequently, the membranes were added with MAbs and incubated at room temperature for 1 h and washed 3 times in PBST. Thereafter, the membranes were incubated with HRP-conjugated anti-mouse IgG (dilution, 1:10000). After washing, N protein signals were detected with chemiluminescence (ECL) detection system (ChemiDoc MP, Bio-Rad Laboratories, Inc., USA).

### Establishment and optimization of the bELISA.

The purified MAb was labeled with horseradish peroxidase (HRP) using HRP conjugation kit according to manufacturer’s instruction (Sigma-Aldrich, St. Louis, MO, USA). To determine the optimal concentrations of the coating antigen and dilutions of the serum, the checkerboard titrations were used. The purified rN was serially diluted (4, 2, 1, 0.5, 0.25, 0.125, 0.0625 and 0.03125 μg/mL) in carbonated coating buffer (pH 9.6), and 50 μL was horizontally added into ELISA plates and incubated at 4°C overnight. The plates were washed 4 times with PBST, and then blocked with 2% (wt/vol) trehalose (100 μL/well) in PBST at 37°C for 1 h. SADS-CoV-positive or negative pig serum were diluted at 1:2, 1:4, 1:8, 1:16, and 1:32 in PBST, and longitudinally added to the wells (50 μL/well), then incubated at 37°C for 30 min. After washing with PBST, 50 μL of HRP labeled MAb (dilution, 1:8000) was added each well, and incubated at 37°C for 30 min. The plates were washed again and developed with TMB (50 μL/well) at 37°C for 10 min, stopped by using 2 M H_2_SO_4_ (50 μL/well). The absorbance was measured at 450 nm. The optimal concentrations of the coating antigen and dilutions of the serum were determined by the highest percent inhibition (PI) values. The PI values were calculated using the following formula: PI (%) = (1-OD_450_ nm of test serum samples/OD_450_ nm of negative-control serum samples) ×100%.

After the conditions mentioned above were determined, the optimal concentration of rN was coated at 4°C overnight. The blocking conditions were optimized as follows. The rN-coated plates were blocked with 5% (wt/vol) skimmed milk, 5% (wt/vol) BSA, or 2%(wt/vol) trehalose in PBST at 37°C for 1 h. To determine the optimal blocking time, the rN-coated plates were blocked in the optimal blocking solution at 37°C for 1, 2, 3, and 4 h, or 4°C for 12 h. Next, the optimal dilution of serum was added and incubated at 37°C for 15, 30, 45, or 60 min. The HRP-MAb was diluted 1:1000, 1:2000, 1:4000, 1:8000, 1:16000, 1:32000, 1:64000, and 1:128000 in PBST, and incubated at 37°C for 30 min. After that, the incubation time of the HRP-MAb were optimized at 37°C for 15, 30, 45, or 60 min. The reactions were stopped and optimized by assessing 5, 10, 15, and 20 min, respectively. All experiments were assessed by the PI values.

### Determination of the cutoff value of the bELISA.

In accordance with the above protocol, 129 serum samples (50 experimental samples and 79 clinical samples) were selected to determine an optimal cutoff value. A receiver operating characteristic (ROC) curve of the PI values was constructed using PASW Statistics for Windows, version 23 (SPSS, Inc., Chicago, IL, USA). The Youden index was calculated as follows: sensitivity-(1-specificity). The tangent point with the largest Youden index was designated the cutoff value.

### Specificity, sensitivity, reproducibility, and stability.

To test the analytical specificity of the bELISA for SADS-CoV, PEDV, TGEV, PDCoV, PoRV, and PSV samples would be the test samples (false positives) and the known SADS-CoV samples would be the assay controls (true positives). Each group contained three samples.

To evaluate the analytical sensitivity of the bELISA for SADS-CoV, the positive and negative standard samples was double diluted to 1:2, 1:4, 1:8, 1:16, 1:32, 1:64, 1:128, 1:256, 1:512, 1:1024, 1:2048, and 1:4096, respectively, and the PI value was calculated.

To test the intraassay reproducibility, serum positive samples P1-6 (including 2 weak positive, 2 midrange positive, and 2 strong positive) and negative of N1-3 (including 1 weak negative, 1 midrange negative, and 1 strong negative) for SADS-CoV were assayed 5 times. Three batches of the bELISA were used to confirm interassay reproducibility. The average value of PI ($\bar{X}$) and the SD values were calculated. The coefficient of variation (CV) was calculated as $(\mathrm{SD}/\bar{X})\times 100\%$.

To determine the stability of the bELISA for SADS-CoV, the ELISA plates, HRP-labeled MAb, 25×PBST, TMB, 2 M H_2_SO_4_, three positive serum samples (P1-3, including 1 weak positive, 1 midrange positive, and 1 strong positive) and six negative serum samples (N1-6, including 1 weak negative and 5 strong negative) stored at 4°C for 0 to 15 months. The ELISA plates were assayed at 0, 1, 2, 3, 6, 9, 12 and 15 months, respectively. 25×PBST was diluted with water to 1×, and other reagents returned to room temperature when used.

### Sample detection.

The 150 clinical serum sample were obtained from different pig farms in Gansu Province, and simultaneously detected by both the bELISA and IFA. The consistency of the bELISA and IFA was evaluated by the total coincidence rate (positive and negative) and kappa value (Κ).

### Ethical statement.

All of the animal experiments were conducted in accordance with the regulations for the administration of affairs concerning experimental animals approved by the State Science and Technology Commission of the People's Republic of China.
