# Supplementary material for: A Customized Novel Blocking ELISA for Detection of Bat-Origin Swine Acute Diarrhea Syndrome Coronavirus Infection
Source: Microbiol Spectr. 2023 Jun 5;11(4):e03930-22. doi: 10.1128/spectrum.03930-22 (PMC10434073; doi:10.1128/spectrum.03930-22)
Supplement: Supplemental file 1 — Supplemental material. Download spectrum.03930-22-s0001.pdf, PDF file, 0.5 MB [file spectrum.03930-22-s0001.pdf]

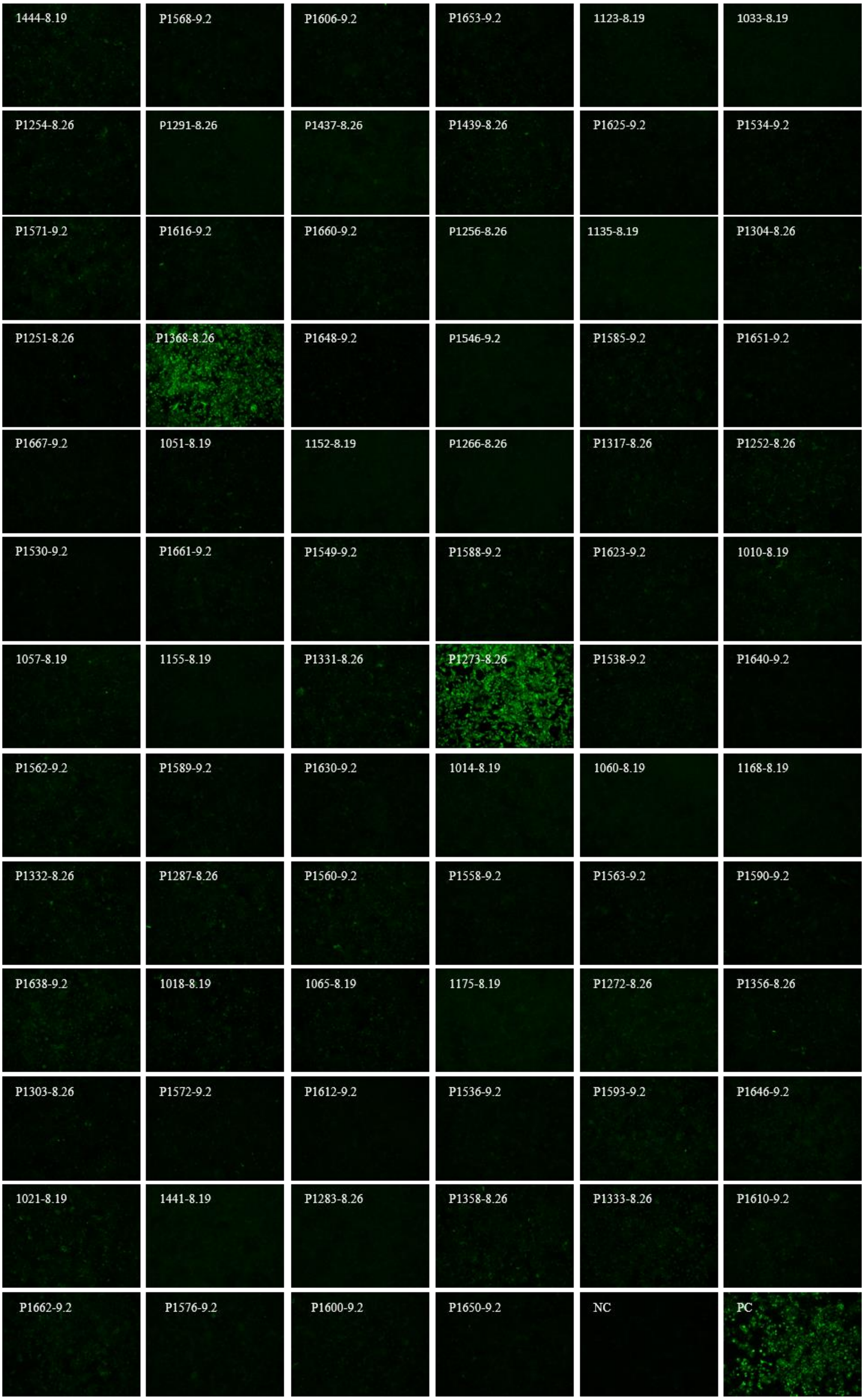
1444-8.19
P1568-9.2
P1606-9.2
P1653-9.2
1123-8.19
1033-8.19
P1254-8.26
P1291-8.26
P1437-8.26
P1439-8.26
P1625-9.2
P1534-9.2
P1571-9.2
P1616-9.2
P1660-9.2
P1256-8.26
1135-8.19
P1304-8.26
P1251-8.26
P1368-8.26
P1648-9.2
P1546-9.2
P1585-9.2
P1651-9.2
P1667-9.2
1051-8.19
1152-8.19
P1266-8.26
P1317-8.26
P1252-8.26
P1530-9.2
P1661-9.2
P1549-9.2
P1588-9.2
P1623-9.2
1010-8.19
1057-8.19
1155-8.19
P1331-8.26
P1273-8.26
P1538-9.2
P1640-9.2
P1562-9.2
P1589-9.2
P1630-9.2
1014-8.19
1060-8.19
1168-8.19
P1332-8.26
P1287-8.26
P1560-9.2
P1558-9.2
P1563-9.2
P1590-9.2
P1638-9.2
1018-8.19
1065-8.19
1175-8.19
P1272-8.26
P1356-8.26
P1303-8.26
P1572-9.2
P1612-9.2
P1536-9.2
P1593-9.2
P1646-9.2
1021-8.19
1441-8.19
P1283-8.26
P1358-8.26
P1333-8.26
P1610-9.2
P1662-9.2
P1576-9.2
P1600-9.2
P1650-9.2
NC
PC

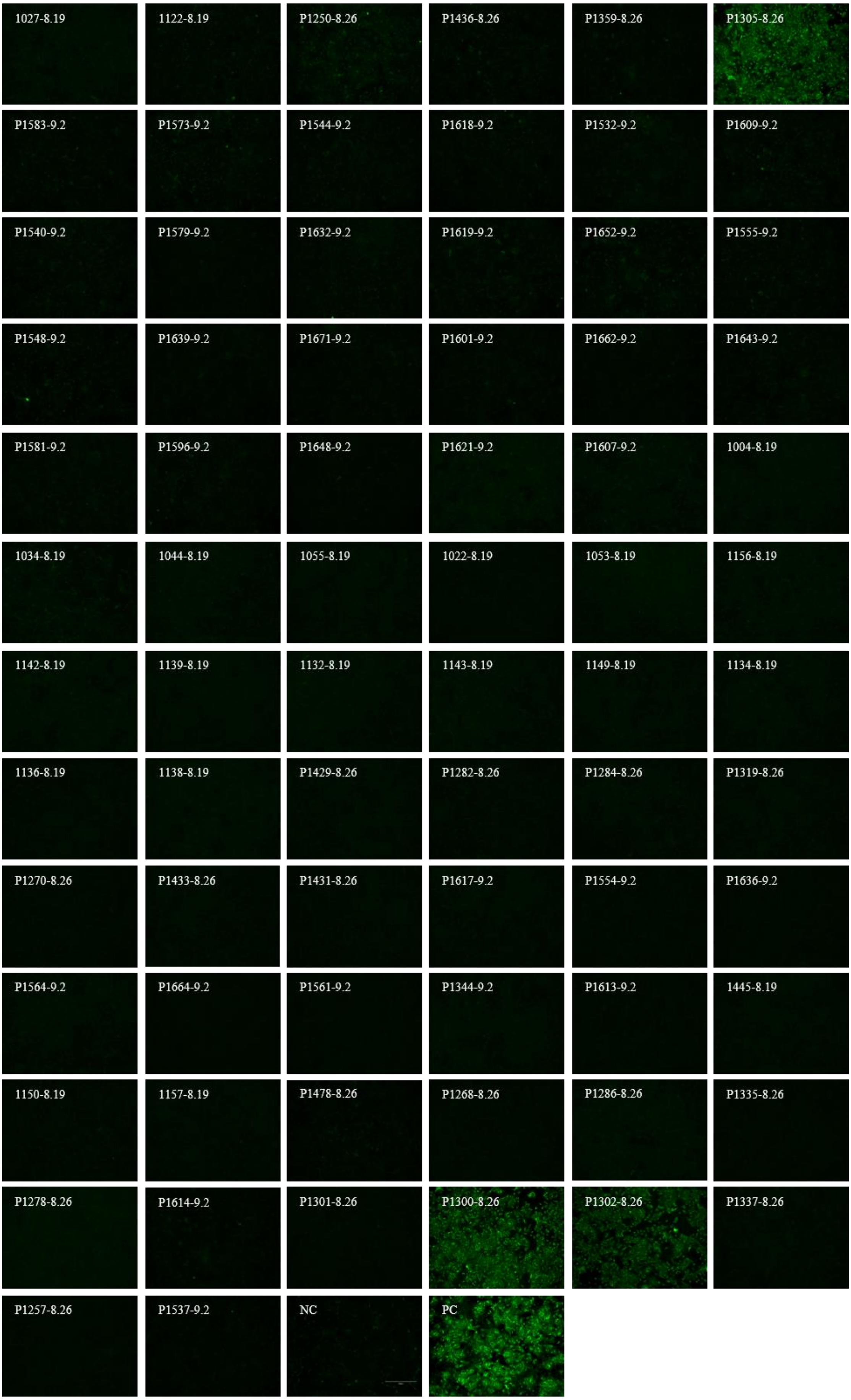
1027-8.19
1122-8.19
P1250-8.26
P1436-8.26
P1359-8.26
P1305-8.26
P1583-9.2
P1573-9.2
P1544-9.2
P1618-9.2
P1532-9.2
P1609-9.2
P1540-9.2
P1579-9.2
P1632-9.2
P1619-9.2
P1652-9.2
P1555-9.2
P1548-9.2
P1639-9.2
P1671-9.2
P1601-9.2
P1662-9.2
P1643-9.2
P1581-9.2
P1596-9.2
P1648-9.2
P1621-9.2
P1607-9.2
1004-8.19
1034-8.19
1044-8.19
1055-8.19
1022-8.19
1053-8.19
1156-8.19
1142-8.19
1139-8.19
1132-8.19
1143-8.19
1149-8.19
1134-8.19
1136-8.19
1138-8.19
P1429-8.26
P1282-8.26
P1284-8.26
P1319-8.26
P1270-8.26
P1433-8.26
P1431-8.26
P1617-9.2
P1554-9.2
P1636-9.2
P1564-9.2
P1664-9.2
P1561-9.2
P1344-9.2
P1613-9.2
1445-8.19
1150-8.19
1157-8.19
P1478-8.26
P1268-8.26
P1286-8.26
P1335-8.26
P1278-8.26
P1614-9.2
P1301-8.26
P1300-8.26
P1302-8.26
P1337-8.26
P1257-8.26
P1537-9.2
NC
PC

Fig. S1 Clinical serum samples were analyzed by IFA. The pig serum samples were diluted 1:500 in PBS, and incubated with SADS-CoV infected cells. The healthy pig serum (as negative control, NC) and pig polyclonal antibody to SADS-CoV (as positive control, PC) were used as control.

Table S1 Clinical serum samples were detected by the bELISA.

Sample NO.	1		2		3	
	PI%	Judge	*PI%*	Judge	*PI%*	Judge
1444-8.19	13.83845	-	12.5861	-	14.0263	-
P1568-9.2	32.7489	-	31.62179	-	32.24796	-
P1606-9.2	33.06199	-	33.8134	-	33.62555	-
P1653-9.2	21.85348	-	22.85535	-	23.48153	-
1033-8.19	4.696306	-	4.070131	-	5.32248	-
1123-8.19	5.760802	-	4.884158	-	7.138384	-
P1254-8.26	17.47026	-	17.15717	-	17.9712	-
P1291-8.26	32.12273	-	30.87038	-	32.43582	-
P1437-8.26	12.9618	-	18.7226	-	13.77583	-
P1439-8.26	32.06011	-	30.43206	-	32.56105	-
P1625-9.2	26.86287	-	27.36381	-	27.42642	-
P1534-9.2	18.15905	-	19.4114	-	17.84596	-
P1571-9.2	13.77583	-	19.16093	-	17.53287	-
P1616-9.2	18.7226	-	19.09831	-	17.9712	-
P1660-9.2	18.47214	-	17.34502	-	19.66187	-
1135-8.19	13.46274	-	15.90482	-	18.40952	-
P1256-8.26	12.08516	-	13.65059	-	16.78147	-
P1304-8.26	27.67689	-	25.42267	-	26.73763	-
P1251-8.26	25.5479	-	26.9881	-	26.36193	-
P1368-8.26	60.59603	+	63.68653	+	61.42384	+
P1648-9.2	9.267376	-	11.89731	-	12.52348	-
P1546-9.2	9.392611	-	9.517846	-	5.572949	-
P1585-9.2	12.83657	-	24.85911	-	29.68065	-
P1651-9.2	12.71133	-	13.33751	-	12.39825	-
P1667-9.2	11.39637	-	10.39449	-	8.70382	-
1051-8.19	3.193488	-	2.191609	-	3.694427	-
1152-8.19	17.84596	-	16.65623	-	16.96932	-
P1266-8.26	13.27489	-	12.21039	-	13.40013	-
P1317-8.26	28.0526	-	23.79461	-	29.55542	-
P1252-8.26	23.60676	-	24.29555	-	23.98247	-
P1530-9.2	34.25172	-	31.05823	-	28.0526	-
P1661-9.2	12.83657	-	-86875.6	-	11.45899	-
P1549-9.2	9.768316	-	14.90294	-	10.20664	-
P1588-9.2	18.97307	-	21.72824	-	22.98059	-
P1623-9.2	-3.44396	-	-2.31684	-	0.876644	-
1010-8.19	7.889793	-	9.89355	-	7.827176	-
1057-8.19	16.59361	-	17.53287	-	12.83657	-
1155-8.19	3.38134	-	9.768316	-	6.073889	-
P1331-8.26	18.47214	-	17.72073	-	18.53475	-
P1273-8.26	48.56512	+	49.06181	+	47.95806	+

Sample NO.	1		2		3	
	PI%	Judge	*PI%*	Judge	*PI%*	Judge
P1538-9.2	11.77207	-	12.21039	-	11.58422	-
P1640-9.2	11.77207	-	11.64684	-	11.33375	-
P1562-9.2	9.89355	-	7.075767	-	7.514089	-
P1589-9.2	24.35817	-	23.04321	-	22.60488	-
P1630-9.2	13.65059	-	18.40952	-	15.40388	-
1014-8.19	13.52536	-	17.90858	-	17.78334	-
1060-8.19	10.51972	-	9.392611	-	10.89543	-
1168-8.19	10.64496	-	10.95805	-	7.889793	-
P1332-8.26	32.99937	-	35.69192	-	36.6938	-
P1287-8.26	22.85535	-	19.91234	-	19.97495	-
P1560-9.2	17.15717	-	14.46462	-	14.27677	-
P1558-9.2	10.01879	-	9.643081	-	9.705698	-
P1563-9.2	15.0908	-	14.77771	-	12.33563	-
P1590-9.2	4.445836	-	2.379461	-	1.94114	-
P1638-9.2	10.83281	-	7.827176	-	7.451472	-
1018-8.19	20.10019	-	20.53851	-	24.98435	-
1065-8.19	5.009393	-	5.886036	-	8.077646	-
1175-8.19	-1.56544	-	-5.7608	-	-3.50657	-
P1272-8.26	15.02818	-	16.78147	-	16.96932	-
P1356-8.26	19.53663	-	17.84596	-	18.84784	-
P1303-8.26	23.16844	-	20.03757	-	24.1077	-
P1572-9.2	12.08516	-	8.891672	-	12.14778	-
P1612-9.2	14.71509	-	12.89919	-	17.65811	-
P1536-9.2	11.1459	-	8.641202	-	9.016907	-
P1593-9.2	5.948654	-	2.316844	-	5.385097	-
P1646-9.2	15.77959	-	13.27489	-	16.71885	-
1021-8.19	14.0263	-	16.65623	-	12.71133	-
1441-8.19	13.14966	-	9.89355	-	12.39825	-
P1283-8.26	-4.00751	-	-0.31309	-	-0.75141	-
P1358-8.26	-4.3206	-	-5.57295	-	-2.44208	-
P1333-8.26	19.47401	-	21.47777	-	23.23106	-
P1610-9.2	17.21979	-	18.91046	-	16.84408	-
P1662-9.2	17.2824	-	16.21791	-	16.78147	-
P1576-9.2	31.05823	-	30.05636	-	30.18159	-
P1600-9.2	2.755166	-	1.127113	-	3.443957	-
P1650-9.2	25.67314	-	25.36005	-	26.6124	-
1027-8.19	2.379461	-	3.819662	-	0.187852	-
1122-8.19	10.64496	-	10.89543	-	12.08516	-
P1250-8.26	32.56105	-	35.31622	-	31.24609	-
P1436-8.26	7.263619	-	12.14778	-	9.830933	-
P1359-8.26	21.60301	-	23.10582	-	20.22542	-
P1305-8.26	61.36865	+	60.09934	+	60.76159	+

Sample NO.	1		2		3	
	PI%	Judge	*PI%*	Judge	*PI%*	Judge
P1583-9.2	24.42079	-	23.66938	-	21.41515	-
P1573-9.2	21.2273	-	20.03757	-	20.97683	-
P1544-9.2	15.56291	-	14.45916	-	14.23841	-
P1618-9.2	24.00662	-	24.88962	-	24.77925	-
P1532-9.2	11.09272	-	6.677704	-	9.933775	-
P1609-9.2	26.71082	-	25.93819	-	25.82781	-
P1540-9.2	22.07506	-	22.51656	-	21.19205	-
P1579-9.2	26.04857	-	27.31788	-	29.35982	-
P1632-9.2	26.65563	-	27.26269	-	25.55188	-
P1619-9.2	15.12141	-	12.85872	-	14.2936	-
P1652-9.2	12.63797	-	14.95585	-	14.23841	-
P1555-9.2	20.75055	-	22.35099	-	19.81236	-
P1548-9.2	29.4702	-	31.73289	-	30.46358	-
P1639-9.2	29.35982	-	31.29139	-	28.47682	-
P1671-9.2	28.09051	-	24.50331	-	26.04857	-
P1601-9.2	13.13466	-	13.8521	-	13.13466	-
P1662-9.2	24.117	-	26.21413	-	26.98675	-
P1643-9.2	32.33996	-	32.28477	-	30.73951	-
P1581-9.2	18.7638	-	22.51656	-	19.31567	-
P1596-9.2	37.36203	-	36.86534	-	37.30684	-
P1648-9.2	29.96689	-	29.13907	-	32.33996	-
P1621-9.2	25.55188	-	26.04857	-	32.22958	-
P1607-9.2	21.90949	-	19.81236	-	22.79249	-
1004-8.19	10.43046	-	8.222958	-	7.560706	-
1034-8.19	20.41943	-	18.3223	-	20.41943	-
1044-8.19	23.6755	-	20.14349	-	18.7638	-
1055-8.19	12.14128	-	11.20309	-	11.47903	-
1022-8.19	10.98234	-	9.437086	-	11.47903	-
1053-8.19	20.41943	-	19.53642	-	22.84768	-
1156-8.19	25.33113	-	27.26269	-	25.27594	-
1142-8.19	3.256071	-	3.532009	-	6.622517	-
1139-8.19	8.664459	-	6.843267	-	10.48565	-
1132-8.19	13.24503	-	16.72185	-	15.72848	-
1143-8.19	21.35762	-	18.48786	-	18.81898	-
1149-8.19	6.181015	-	6.677704	-	4.690949	-
1134-8.19	21.46799	-	21.90949	-	17.88079	-
1136-8.19	22.73731	-	22.40618	-	21.19205	-
1138-8.19	8.719647	-	10.37528	-	6.898455	-
P1429-8.26	0.055188	-	-2.53863	-	-3.31126	-
P1282-8.26	-8.27815	-	-6.95364	-	-8.66446	-
P1284-8.26	6.512141	-	10.2649	-	7.395143	-
P1391-8.26	-0.11038	-	-3.91832	-	-2.37307	-

Sample NO.	1		2		3	
	PI%	Judge	*PI%*	Judge	*PI%*	Judge
P1207-8.26	6.125828	-	4.801325	-	1.710817	-
P1433-8.26	8.057395	-	10.92715	-	6.953642	-
P1431-8.26	-7.78146	-	-7.56071	-	-8.88521	-
P1617-9.2	10.04415	-	10.92715	-	8.774834	-
P1554-9.2	4.19426	-	3.256071	-	0.93819	-
P1636-9.2	11.47903	-	12.63797	-	11.53422	-
P1564-9.2	20.03311	-	18.7638	-	21.90949	-
P1664-9.2	23.234	-	23.17881	-	25.33113	-
P1561-9.2	8.222958	-	6.181015	-	11.09272	-
P1334-9.2	28.25607	-	28.20088	-	26.60044	-
P1613-9.2	30.46358	-	28.75276	-	28.5872	-
1445-8.19	23.06843	-	27.59382	-	28.80795	-
1150-8.19	15.78366	-	12.96909	-	11.09272	-
1157-8.19	22.84768	-	26.26932	-	23.95143	-
P1478-8.26	4.070131	-	3.63181	-	1.628053	-
P1268-8.26	33.11258	-	33.0574	-	31.45695	-
P1286-8.26	28.74139	-	26.9881	-	25.42267	-
P1335-8.26	6.291390	-	7.781457	-	8.057395	-
P1278-8.26	33.99558	-	35.81678	-	34.98896	-
P1614-9.2	18.03381	-	15.0908	-	17.15717	-
P1301-8.26	30.7947	-	32.22958	-	32.28477	-
P1300-8.26	65.34216	+	63.90728	+	62.30684	+
P1302-8.26	50.16556	+	53.42163	+	52.15232	+
P1337-8.26	54.96689	+	54.58057	+	58.27815	+
P1257-8.26	31.34658	-	30.7947	-	31.6777	-
P1537-9.2	30.57395	-	27.53863	-	28.69757	-

“1, 2, and 3” were the different batches of blocking ELISA plates. “+” was positive, and “-” was negative.
